# Childhood brain size mediates the relationship between birthweight and early‐life cognitive ability

**DOI:** 10.1002/alz70860_098001

**Published:** 2025-12-23

**Authors:** Scott T Chiesa, Sarah‐Naomi James, Holly T Haines, Sana Suri, Alun D Hughes

**Affiliations:** ^1^ UCL Unit for Lifelong Health and Ageing, London, England, United Kingdom; ^2^ University College London, London, England, United Kingdom; ^3^ University of Oxford, Oxford, England, United Kingdom; ^4^ UCL Unit for Lifelong Health & Ageing, London, England, United Kingdom

## Abstract

**Background:**

Low cognitive ability in the early decades of life is associated with an increased risk of dementia in old age. Weight and head size are closely correlated developmental markers routinely measured at birth and during infancy and have both been shown to associate with cognitive development in early childhood. We aimed to 1) test whether these associations remained during the transition to adolescence and 2) identify potential mediating pathways linking these factors in the interim years.

**Method:**

Data from 4,896 participants born at a gestational age ≥ 37 weeks and with birthweight ≥ 2.5kg were assessed within the Avon Longitudinal Study of Parents and Children (ALSPAC). Weight and head circumference (as a proxy for brain size) were measured at birth, 8, and 15 years. IQ (as a proxy for early cognitive reserve) was derived at 4, 8, and 15 years. Linear regression and structural equation models (SEM) were used assess interrelationships between weight, head circumference and cognitive function across childhood and adolescence while controlling for gestational age, sex, parent's education, and household social class.

**Result:**

Regression analyses demonstrated evidence for independent associations between both weight and head circumference at birth with IQ at ages 4, 8, and 15. Effect sizes between birthweight and IQ weakened with the passing of time (SD‐difference [95%CI]=0.14[0.01,0.27] at age 4 to 0.04[0.00,0.09] at age 15), but remained consistent for birth head circumference (∼0.10 throughout). SEM identified multiple indirect pathways linking head circumference at birth to IQ in adolescence, all operating via early differences in childhood IQ and head size by the age of 8 years. In contrast, no pathways linking birthweight to any measure of IQ were identified after accounting for accompanying covariance with head size.

**Conclusion:**

Early differences in brain size and IQ established during the formative years of life likely explain much of the association linking early‐life weight status to later cognitive ability and reserve.

